# Supplementary material for: Giant anisotropic magnetoresistance in a quantum anomalous Hall insulator
Source: Nat Commun. 2015 Jul 7;6:7434. doi: 10.1038/ncomms8434 (PMC4507013; doi:10.1038/ncomms8434)
Supplement: Supplementary Information — Supplementary Figures 1-12, Supplementary Notes 1-5 and Supplementary References. [file ncomms8434-s1.pdf]

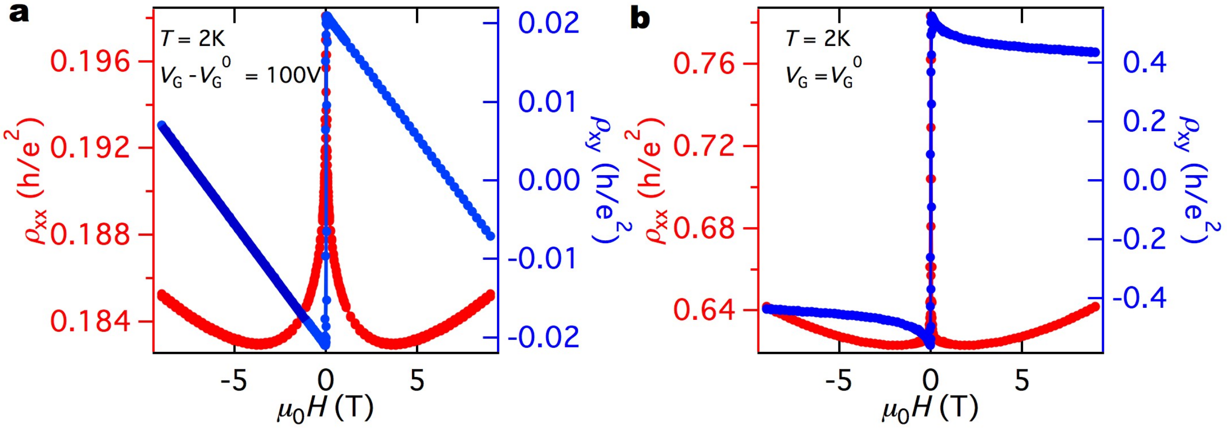

FIG. 1: **(Supplementary Figure 1: Large-field Hall data) (a)** AHE (blue) and longitudinal MR (red) of device A at T =2 K and V_G- $V_G{}^0$ = 100 V. Bold blue line is linear fit to large field Hall data (larger than 1T) **(b)** AHE (blue) longitudinal MR (red) at T =2K and V_G= $V_G{}^0$. The large non-linearity in the large field Hall data makes it difficult to extract reasonable numbers for the carrier density.

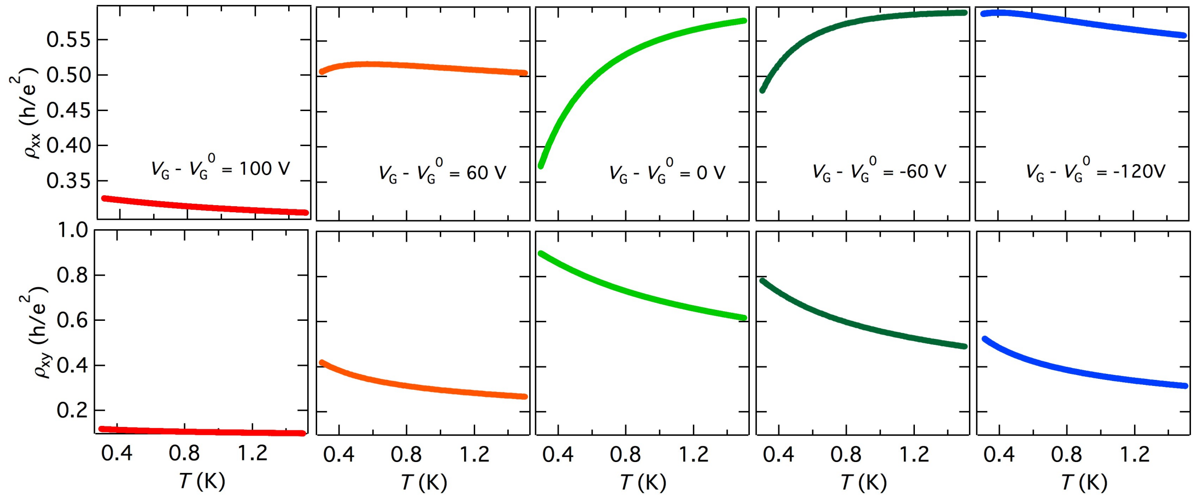

FIG. 2: **(Supplementary Figure 2: Evidence of edge-state transport I)** The data in this figure is taken in an external out-of-plane field of magnitude 1 T. Temperature dependence of ρ_{xx} (top) and ρ_{xy} (bottom) for V_G - $V_G{}^0$ = -120 V (blue), -60 V (dark green), 0 V (light green), 60 V (orange), 100 V (red)

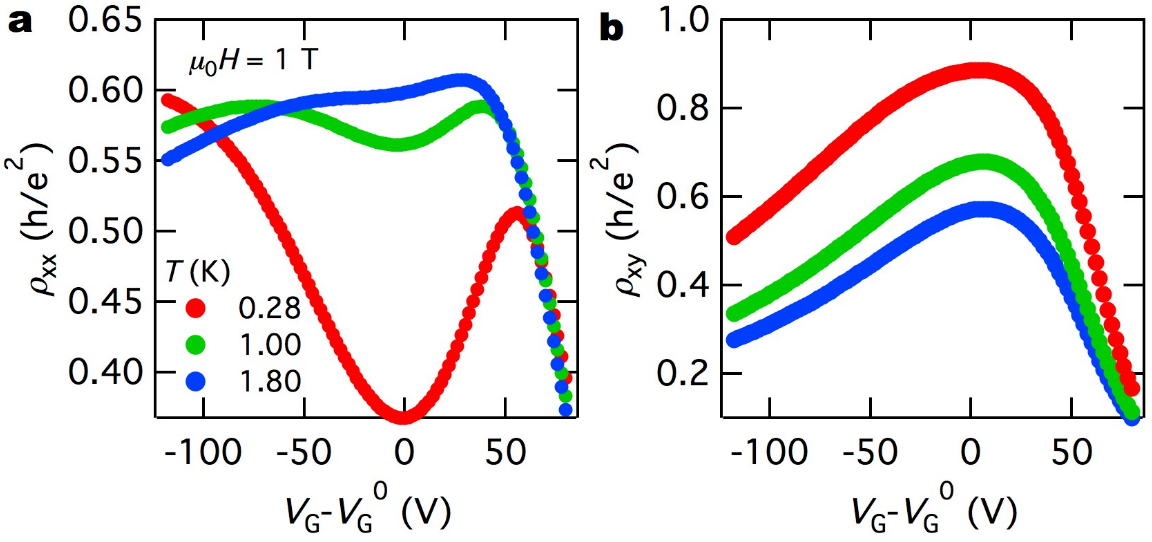

FIG. 3: **(Supplementary Figure 3: Evidence of edge-state transport II)** The data in this figure is taken in an external out-of-plane field of magnitude 1 T. Gate voltage dependence at 280 mK of $\rho_{\rm xx}$ (a) and $\rho_{\rm xy}$ (b) at T = 0.28 K (red), 1.0 K (blue), 1.6 K (light green)

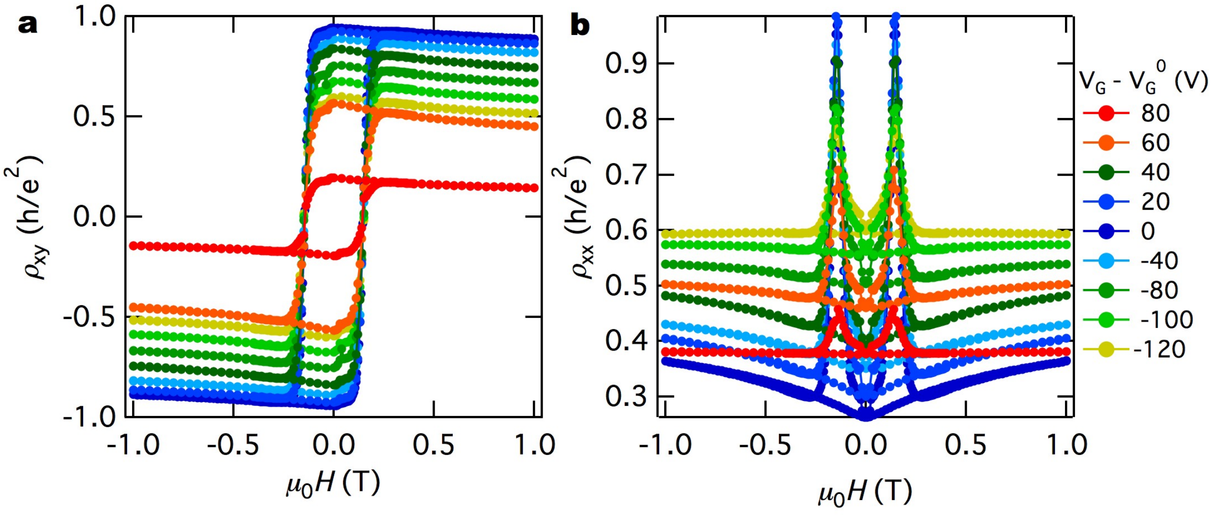

FIG. 4: **(Supplementary Figure 4: Gate dependence of AHE)** **(a)** Longitudinal MR curves for perpendicular-to-plane field sweeps for a range of gate voltages, at T = 280 mK **(b)** Corresponding AHE curves.

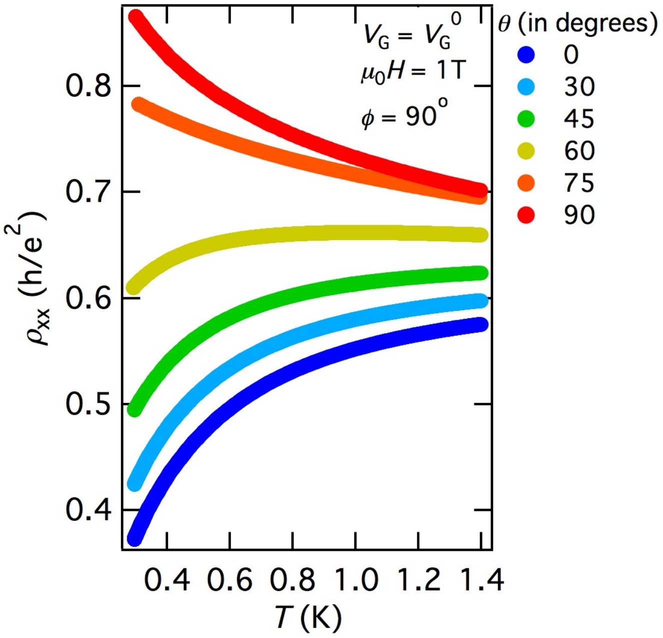

FIG. 5: **(Supplementary Figure 5: Metal-insulator transition)** Temperature dependence of ρ_{xx}, for a range of polar angles as a 1T field is tilted in the $x - z$ plane, all at $V_{\mathrm{G}}= V_{\mathrm{G}}{}^0$.

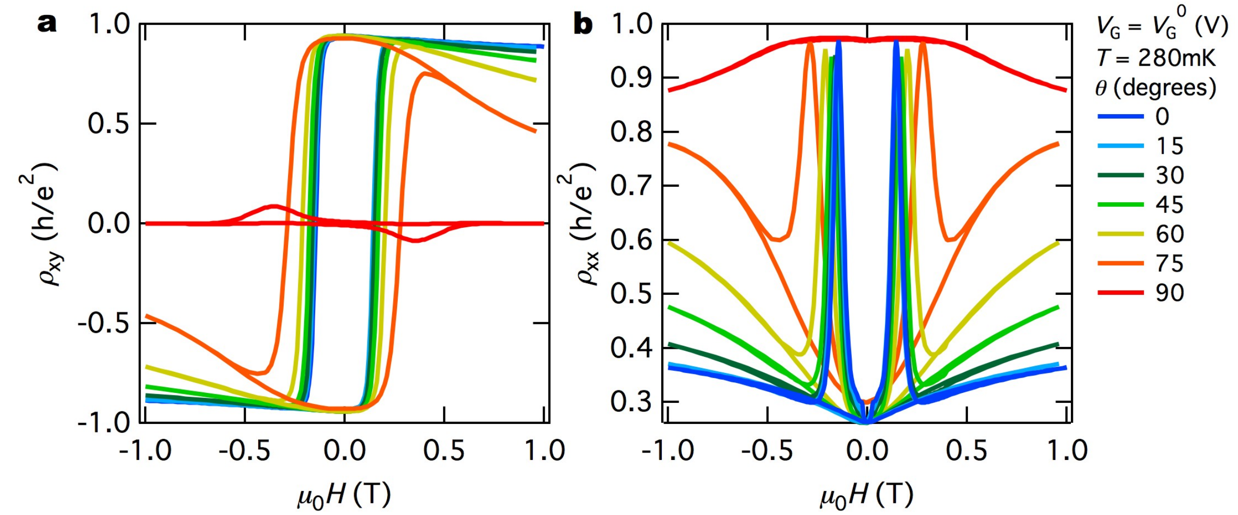

FIG. 6: **(Supplementary Figure 6: Out-of-plane field sweeps)** Field sweeps of **(a)** ρ_{xx} and **(a)** ρ_{xy} for different polar angles in the $x - z$ plane, at T=280 mK and $V_{\mathrm{G}} = V_{\mathrm{G}}{}^0$. The field is tilted from perpendicular-to-plane ($\theta = 0^o$, bottom curve) to in-plane, parallel to current ($\theta = 90^o$, top curve) in steps of 15°. The curves are not offset.

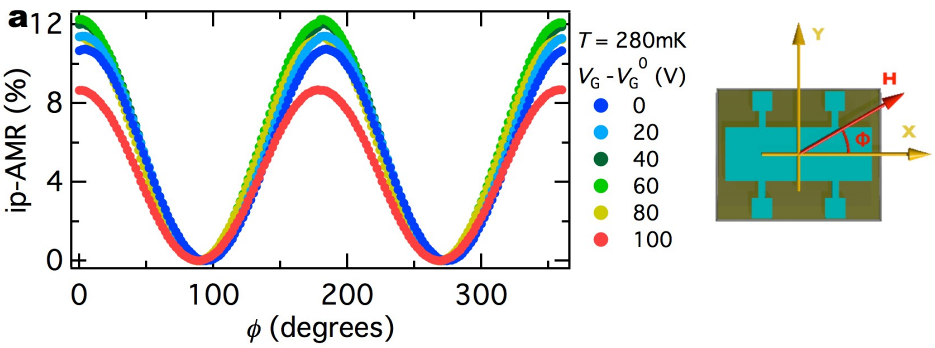

FIG. 7: **(Supplementary Figure 7: In-plane AMR)** **(a)** In-plane angular sweeps at different gate voltages, for a 1T field rotation at 280 mK.

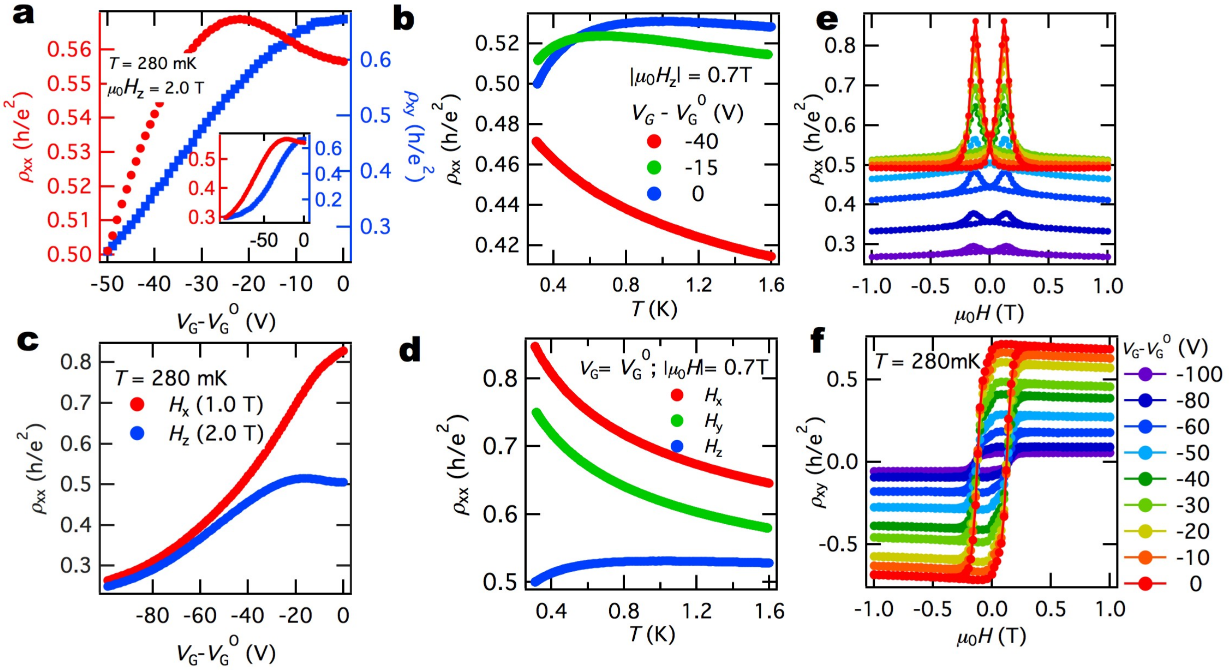

FIG. 8: **(Supplementary Figure 8: Device B characterization)** **(a)** Gate dependence of ρ_{xx} (red) and ρ_{xy} (blue) at 280 mK, in a 2 T perpendicular-to-plane field. Inset: Gate dependence over the entire field range. **(b)** Temperature dependence of ρ_{xx} at V_G -$V_G{}^0$ = -40 V (red), 15 V(green) and 0 V(blue), with the film magnetized out of plane. **(c)** Gate dependence of ρ_{xx} at 280 mK, with the film magnetized along the z axis (blue) and x axis (red) **(d)** Temeperature dependence of ρ_{xx} at charge neutrality, for magnetization along the x (red), y (green) and z (blue) axes.**(e)** Gate dependence of longitudinal MR at 280 mK **(f)** Corresponding gate dependence of AHE.

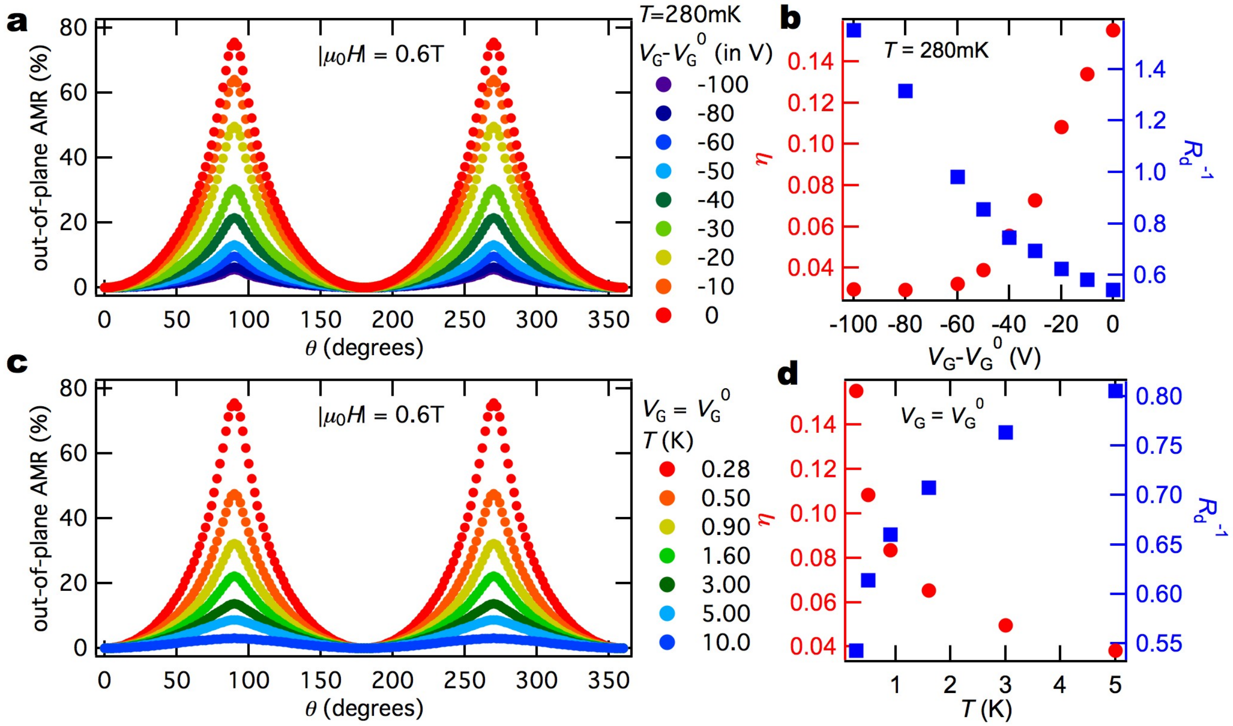

FIG. 9: **(Supplementary Figure 9: Device B AMR)** **(a)** Out of plane AMR at 280 mK for a range of gate voltages, as a 0.6 T field is rotated in the $x - z$ plane. **(b)** Gate voltage dependence of the edge-state transmission coefficient η (red) and the dissipative transmission coefficient $R_d^{-}1$ (blue) at 280 mK, extracted from fits to the data of (a). **(c)** Out-of-plane AMR at $V_{\mathrm{G}}{}^0$ for a range of temperatures. **(d)** Temperature dependence of η (red) and $R_d^{-}1$ (blue) at $V_{\mathrm{G}} = V_{\mathrm{G}}{}^0$ extracted from fits to the data of (c).

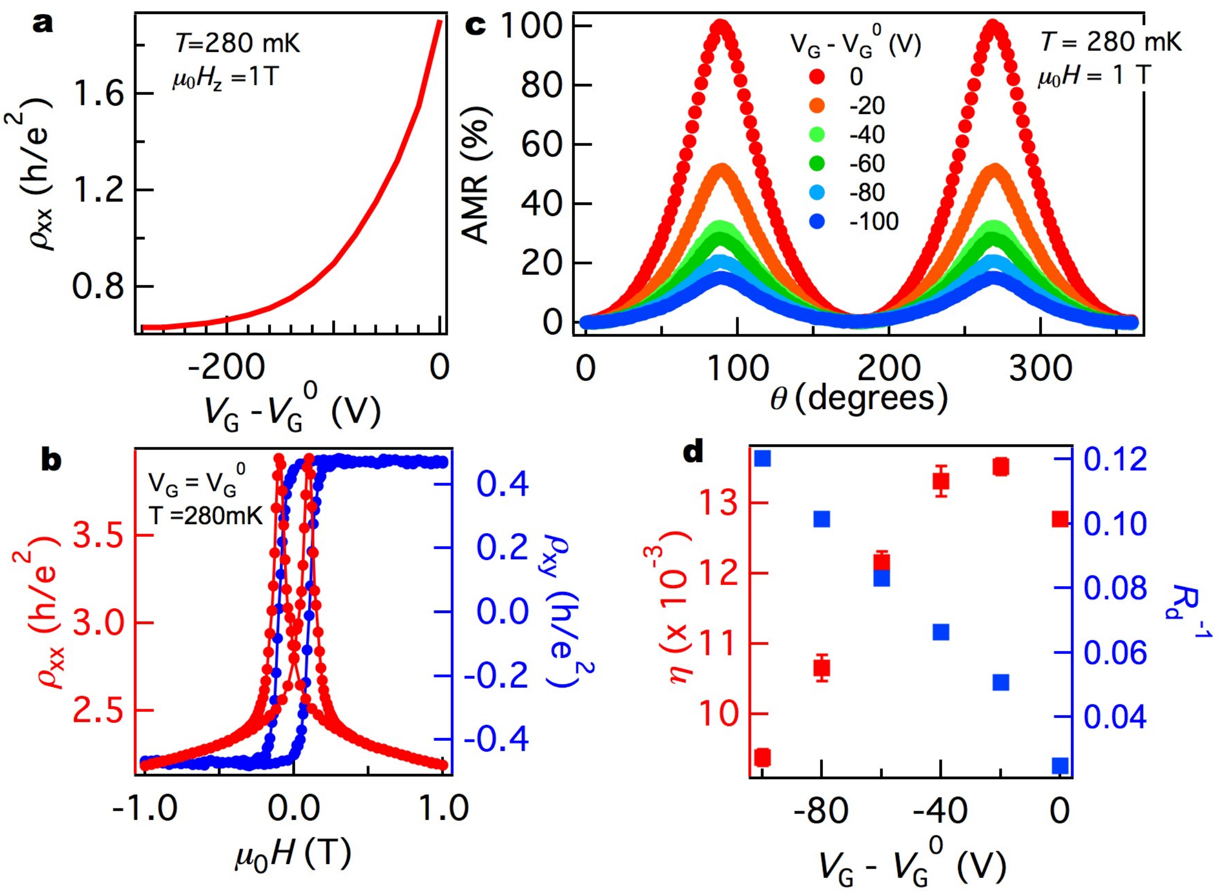

FIG. 10: **(Supplementary Figure 10: Device C)** **(a)** Gate dependence of $\rho_{\rm xx}$ at 280mK, in a 1T perpendicular-to-plane field.**(b)** Longitudinal MR (red) and AHE (blue) at 280 mK, at $V_{\rm G} = {V_{\rm G}}^0$. **(c)** Out-of-plane AMR at 280 mK for a range of gate voltages, as a 1T field is rotated in the $x-z$ plane. **(d)** Gate voltage dependence of η (red) and R_d^-1 (blue) at 280 mK extracted from fits to the data of (c).

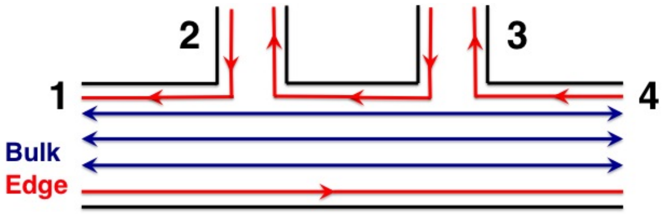

FIG. 11: **(Supplementary Figure 11: Model Cartoon)** Diagrammatic representation of the simplistic four-terminal geometry employed for the edge-dissipation mixing model. The red lines depict the chiral edge modes, while the blue lines depict dissipation channels.

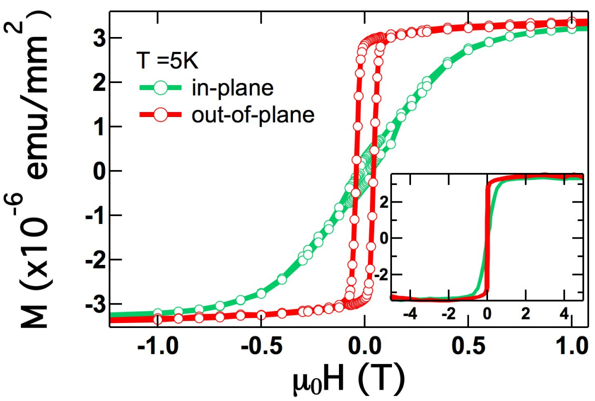

FIG. 12: **(Supplementary Figure 12: SQUID magnetometry)** M v/s $\mu_0 H$ loops of a 100 QL film of $Cr_x(Bi,Sb)_{2-x}Te_3$ on STO at 5 K, for external field applied perpendicular to plane (red) and in-plane (green). The inset shows the M v/s $\mu_0 H$ data over a 5 T field range.

Supplementary Note 1 — Device characterization: Device A

Device A is patterned from a 10 QL film of $Cr_{0.1}(Bi_{0.5},Sb_{0.5})_{1.9}Te_3$, grown on STO, and capped with a thin film of Al that naturally oxidizes upon exposure to atmosphere. We note that for $Cr_x(Bi,Sb)_{2-x}Te_3$ films of this thickness, the top and bottom surfaces are not hybridized.[1].

Large-field Hall measurements

The large non-linearity in the Hall resistivity ρ_{xy} beyond the coercive field, particularly when gated near charge neutrality, makes it difficult to extract accurate numbers for carrier concentration and mobility. This can be seen in the large field Hall data for the main device of the paper, device A, shown in Supplementary Figure 1. These are measurements performed in a Quantum Design PPMS system with a 9T superconducting magnet. We attempt to extract some approximate numbers for the Hall resistivity at large fields in Supplementary Figure 1 (beyond 1T). For a ferromagnet, the transverse resistivity is given by $\rho_{\mathrm{xy}} = R_H H + M f(\rho_{xx})$ where R_H is the Hall resistance, H is the external applied field, M is the magnetization, and $f(\rho_{xx})$ is some function of the longitudinal resistivity. In this system, for the field range above 1T, the magnetization has reached saturation, and the percentage change in ρ_{xx} is very small. Therefore, one can assume the field dependence of the second term in the above expression to be very small, and approximate it to be a constant. Now, by a simplistic linear fit to the large field hall we can obtain rough estimates for single carrier density and mobility. When the film is not gated (V_{G}-$V_{\mathrm{G}}{}^0$ = 100V, left panel), we extract a 2D electron density $n = 7.7\times10^{12}$ cm^{-2}, and a mobility $\mu = 161.44$ cm^2/Vs. The observation of edge state transport despite such low mobilities is one of the remarkable properties of the QAHE.

Evidence of edge state transport: Gate and temperature dependence of ρ_{xx}

The drop in ρ_{xx}, as ρ_{xy} continues to rise, both in their temperature dependence (Supplementary Figure 2) and gate voltage dependence (Supplementary Figure 3) , are indicative of 1D edge transport dominating over diffusive transport. This metallic temperature dependence of ρ_{xx} is unique to QAH physics. Other magnetically doped (and undoped) topological insulator thin films systems outside the QAH regime have typically shown an insulating behavior in this temperature range, often associated with e-e interactions[2]. Supplementary Figure 2 demonstrates that as dissipative channels are populated by pushing the chemical

potential towards the bulk conduction band, a typical insulating behavior is recovered. Additionally, gate sweeps (Supplementary Figure 3) show that the drop in $\rho_{\rm xx}$, observed as we tune into the magnetic gap, is weakened at higher temperatures. This is possibly due to the increased thermal excitation of dissipative channels.

Gate dependence of AHE

The gate dependence of the AHE and the corresponding longitudinal MR is shown in Supplementary Figure 4. While previous reports have demonstrated the quantum anomalous Hall effect at dilution fridge temperatures, we already observe a zero-field Hall resistivity $\rho_{\rm xy}$ tantalizingly close to quantization ($\sim 0.95h/e^2$), even at 280mK. The squareness of the AHE is clearly indicative of the out-of-plane magnetic anisotropy: an important prerequisite for accessing the QAHE regime. While initial reports of the QAHE achieved a vanishing $\rho_{\rm xx}$ upon application of an external field [3, 4], our device instead shows a rise in resistance at charge neutrality as also reported in thicker $\mathrm{Cr}_x(\mathrm{Bi,Sb})_{2-x}\mathrm{Te}_3$ films [5]. The typical negative MR is however recovered when the chemical potential is tuned towards the conduction band (see curve at $V_{\rm G}$- $V_{\rm G}{}^0 = 80$V, Supplementary Figure 4). The coercive field is seen to be constant over this voltage range.

Angle tuned metal-insulator transition

A systematic study of the transition from metallic to insulating behavior, in the temperature dependence of $\rho_{\rm xx}$, is presented in Supplementary Figure 5. In contrast to the regime of perfect Hall quantization, the temperature scale for observing such signatures of edge transport is much higher, and is set by the position of the chemical potential, the size of the magnetic gap, and/or the thermal activation energy of in-gap impurity bands. In Supplementary Figure 5, the chemical potential of the film is kept fixed at charge neutrality, while the size of the magnetic gap is tuned by changing the strength of the perpendicular component of magnetization. Clearly, metallic behavior sets in at lower temperatures as the magnetization is tilted in-plane, and eventually beyond some critical angle, $\rho_{\rm xx}$ only shows insulating behavior. These results clearly demonstrate magnetization control of edge-transport.

Out-of-plane field sweeps: $x - z$ plane

The field sweeps of Supplementary Figure 6 at different polar angles in the $x - z$ plane confirm that our chosen field of 1T for the AMR measurements is well beyond the field range of any hysteresis. Also, a comparison of the perpendicular-to-plane and in-plane MR

curves reveals the strong out-of-plane magnetic anisotropy. The sharp ρ_{xx} peak in the perpendicular-to-plane sweep may be compared to the low-field $\rho_{xx}$of the in-plane sweep. Both are very similar, since they represent the identical magnetic state of net zero magnetization. Similarly, the AHE curves of Supplementary Figure 6b demonstrate that even for sweeps at large tilt angles (75°), the zero-field remenant state is strongly out-of-plane, leading to a near quantized Hall resistivity. Slight offsets in the in-plane field sweeps lead to significantly large zero-field Hall resistivity. Therefore, we ensure that the deviation in our in-plane sweep is less than 0.1^o.

In-plane AMR

As the chemical potential is tuned from the conduction band towards the magnetic gap, the in-plane AMR shows a weak, non-monotonic gate dependence, unlike the out-of-plane AMR (Supplementary Figure 7). However, the $\cos^2\phi$ angular dependence is unchanged over the entire gating range.

Supplementary Note 2 — Device B

Device characterization: Device B

We present data from Device B, which reproduces the observations of the Device A. A 8QL thin film of $Cr_{0.15}(Bi_{0.48},Sb_{0.52})_{1.85}Te_3$ was mechanically scatched into a Hall bar with channel dimensions 450 μm $\times$ 200 μm. The device shows the essential signatures of edge transport: a drop in ρ_{xx}, accompanied by a rise in ρ_{xy}(Supplementary Figure 8). In contrast to the film of device A, this film is p-type as grown, as one can see in the gate dependence of ρ_{xx}. Below 1K, when the film is at charge neutrality ($V_G{}^0 = 100$V), ρ_{xx} shows a metallic temperature dependence. The onset of metallic behavior depends on the the position of the chemical potential, and if sufficiently far from the magnetic gap, the typical insulating behavior is recovered (Supplementary Figure 8b). These drops in the gate and temperature dependence of ρ_{xx} are destroyed by magnetizing the film in-plane (Supplementary Figure 8c,d). Finally, at 280mK, and charge neutrality ($V_G = V_G{}^0$), the devices shows a maximum AHE of 0.72 h/e^2 and a zero-field ρ_{xx} of 0.55 h/e^2 (Supplementary Figure 8e,f).

Giant AMR and fitting: Device B

An additional data set from device B, presented in Supplementary Figure 9 shows the evolution of the out-of-plane AMR as the chemical potential is tuned from the magnetic gap, into the bulk valence band. This is in contrast to Device A in which we solely accessed the electron transport regime. The functional dependence of the AMR is unchanged even as the chemical potential is tuned into the bulk valence band, and we extract self-consistent fitting parameters over the entire voltage range. This further conveys the effectiveness of our methodology in quantifying edge contributions to transport in temperature and chemical potential regimes far from perfect Hall quantization.

Supplementary Note 3 — Device C

Device C is the farthest from quantization (max. $\rho_{xy}\sim 0.48$ h/e^2) of the devices studied in this work, and is plagued by a very large zero-field $\rho_{xx}\sim 2.79$ h/e^2 (at $V_G{}^0 = 140$V), indicative of large dissipation. The Hall bar was scratched with channel dimensions 1200 μm $\times$ 170 μm, from a 7QL film of $Cr_{0.23}(Bi_{0.39},Sb_{0.61})_{1.77}Te_3$. The gate dependence of ρ_{xx} in Supplementary Figure 10a does not show any signatures of QAH related edge-state transport. However, the measurement (Supplementary Figure 10c) and fitting (Supplementary Figure 10d) of the out-of-plane AMR provide the means to identify and quantify edge-state contributions to transport. Consistent with the film quality, the extracted values of the edge transmission co-efficient η are over an order of magnitude smaller than those extracted for devices A and B.

Supplementary Note 4 — Landauer-Buttiker formalism for quantum anomalous Hall with dissipative channels

We employ a simplistic four-terminal Landauer-Buttiker formulism $I_i = \Sigma_j G_{ij}(V_i - V_j)$ that accounts for edge channels in the presence of dissipation paths. The model geometry is depicted in Supplementary Figure 11. The conductance G_{ij} is given by $G_{ij} = T_{ij}(h/e^2)$ where T_{ij} is the transmission co-efficient. We label the four terminals as 1, 2, 3 and 4 where 2 and 3 are the voltage terminals. The Landauer-Buttiker formula may then be expressed as

$$\begin{pmatrix} I \\ 0 \\ 0 \end{pmatrix} = \begin{pmatrix} G_{12}+G_{13}+G_{14} & -G_{12} & -G_{13} \\ -G_{21} & G_{21}+G_{23}+G_{24} & -G_{23} \\ -G_{31} & -G_{32} & G_{31}+G_{32}+G_{34} \end{pmatrix} \begin{pmatrix} V_1 \\ V_2 \\ V_3 \end{pmatrix} \tag{1}$$

There transmission co-efficient takes the form $T_{ij} = \eta\delta_{i,j+1} + t_{ij}$, where η is the contribution from edge modes that transmit between leads j and $j+1$, and t_{ij} accounts for dissipative contributions. For simplicity, we make the following assumptions: $t_{23} = t_{32} = T_1$ and $t_{12} = t_{21} = t_{34} = t_{43} = T_2$, and neglect transmission for longer contact separations. This reduces the above equation to

$$\begin{pmatrix} I \\ 0 \\ 0 \end{pmatrix} = \begin{pmatrix} T_2+\eta & -T_2 & 0 \\ -(\eta+T_2) & \eta+T_2+T_1 & -T_1 \\ 0 & -(\eta+T_1) & T_1+\eta+T_2 \end{pmatrix} \begin{pmatrix} V_1 \\ V_2 \\ V_3 \end{pmatrix} \tag{2}$$

By multiplying the matrices we obtain

$$V_3 = \frac{(\eta+T_1)V_2}{\eta+T_2+T_1} \tag{3}$$

$$V_1 = \frac{1}{\eta+T_2}((\eta+T_2+T_1)V_2 - T_1V_3) = \frac{1}{\eta+T_2}((\eta+T_2+T_1)V_2 - T_1\frac{(\eta+T_1)V_2}{\eta+T_2+T_1}) \tag{4}$$

$$I = (T_2+\eta)V_1 - T_2V_2 = (\eta+T_1)V_2\frac{\eta+T_2}{\eta+T_2+T_1} \tag{5}$$

The measured longitudinal resistance in units of h/e^2 is then defined as

$$R = \frac{V_2 - V_3}{I} = \frac{V_2}{I}(1 - \frac{\eta+T_1}{\eta+T_2+T_1}) \tag{6}$$

$$R = \left(\frac{\eta(\eta+T_1+T_2)}{T_2} + T_1\right)^{-1} \tag{7}$$

The denominator of the Supplementary equation 7 has two terms, the first of which is associated with the edge states. One can see that in the limit of dominant edge transport, we have $\eta \gg T_1, T_2 \implies \eta/T_2 \to \infty$ and therefore, $R \to 0$ as one would expect for pure chiral edge transport. For the other extreme of no edge transport we have $\eta = 0$, and $R = 1/T_1$. Therefore, the second term in the denominator of Supplementary equation 7 corresponds to solely the dissipative resistance, $R_d = 1/T_1$. Supplementary equation 7 is now re-written as

$$R = \left(\frac{\eta(\eta + R_d^{-1} + T_2)}{T_2} + R_d^{-1} \right)^{-1} \tag{8}$$

With this correlation between the dissipative transmission co-efficient and the dissipative resistance emerging, and based on typical geometric considerations, we now make the assumption $T_1 \approx T_2$. This is reasonable since the two-point resistances of adjacent contacts may be expected to be of the same order. This also serves to reduce the number of free fitting parameters. The longitudinal resistance now reduces to the expression described in the main text after including the h/e^2.

$$R = \frac{h}{e^2} \frac{1}{\eta(2 + R_d \eta) + R_d^{-1}} \tag{9}$$

Supplementary Note 5 — Out-of-plane magnetic anisotropy of $Cr_x(Bi,Sb)_{2-x}Te_3$: SQUID Magnetometry

The large background of the STO substrate and the small thickness of our $Cr_x(Bi,Sb)_{2-x}Te_3$ films used in transport experiments make it difficult hard to extract magnetization hysteresis loops by standard superconducting quantum interfering device (SQUID) magnetometry. Instead, we perform magnetometry on a 100QL thick film of $Cr_x(Bi,Sb)_{2-x}Te_3$ grown on InP in a Quantum Design PPMS system, with a significantly higher $T_c(\sim 60K)$, in order extract clean hysteresis loops. Supplementary Figure 12 shows the M v/s $\mu_0 H$ curves for in-plane and out-of-plane configurations at 5K, and clearly demonstrates that the easy axis of the $Cr_x(Bi,Sb)_{2-x}Te_3$ system lies out-of-plane.

1. Jiang, Y. *et al.* Landau Quantization and the Thickness Limit of Topological Insulator Thin Films of Sb_2Te_3. *Phys. Rev. Lett.* **108**, 016401 (2012).

2. Wang, J. *et al.* Evidence for electron-electron interaction in topological insulator thin films. *Phys. Rev. B* **83**, 245438 (2011).

3. Chang, C.-Z. *et al.* Experimental observation of the quantum anomalous Hall effect in a magnetic topological insulator. *Science* **340**, 167-170 (2013).

4. Checkelsky, J. G. *et al.* Trajectory of the anomalous Hall effect towards the quantized state in a ferromagnetic topological insulator. *Nat. Phys.* **10**, 731-736 (2014).

5. Kou, X., *et al.* Scale-invariant dissipationless chiral transport in magnetic topological insulators beyond two-dimensional limit. *Phys. Rev. Lett.* **113**, 137201 (2014).
